# Optimal Transducer Placement for Deep Learning-Based Non-Destructive Evaluation

**DOI:** 10.3390/s23031349

**Published:** 2023-01-25

**Authors:** Ji-Yun Kim, Je-Heon Han

**Affiliations:** Department of Mechanical Engineering, Tech University of Korea, Siheung-si 15073, Republic of Korea

**Keywords:** deep learning, CNN, non-destructive evaluation, lamb waves, transducer array

## Abstract

In this study, the Convolution Neural Network (CNN) algorithm is applied for non-destructive evaluation of aluminum panels. A method of classifying the locations of defects is proposed by exciting an aluminum panel to generate ultrasonic Lamb waves, measuring data with a sensor array, and then deep learning the characteristics of 2D imaged, reflected waves from defects. For the purpose of a better performance, the optimal excitation location and sensor locations are investigated. To ensure the robustness of the training model and extract the feature effectively, experimental data are collected by slightly changing the excitation frequency and shifting the location of the defect. The high classification accuracy for each defect location can be achieved. It is found that the proposed algorithm is also successfully applied even when a bar is attached to the panel.

## 1. Introduction

Ultrasonic bulk waves are commonly used to identify structural defects by applying “time of flight” measurement-based methods such as the pulse-echo method and the through-transmission method. They have been actively used in the industry because of its wide application and high precision at a relatively low cost. Mouritz et al. [1] evaluated the fatigue damage of polymer-matrix composites used in ships by applying the pulse-echo method. Hao et al. [2] employed a low frequency ultrasonic pulse-echo method to investigate aging of large generators by comparing and analyzing four types of stator bar insulation structures. Tian et al. [3] performed the pulse-echo test to detect void defects in epoxy composite specimens. Lee et al. [4] developed a rotational through-transmission ultrasonic imaging system to identify a cylindrical pressure vessel damage and obtained the clear scanned images. 

In addition to detecting structural defects, the ultrasonic non-destructive testing methods also can be used to measure the thickness [5,6,7] or elasticity [8] of the material. Furthermore, ultrasonic flaw detection technique has been applied to food [9] and medical fields [10,11,12]. As described above, ultrasonic non-destructive inspection methods are capable of detecting defects on several structures and can be used for various purposes.

Since these methods can only inspect a local area, it is inconvenient and time-consuming to scan a region of interest with an ultrasonic probe. For this reason, using guided ultrasonic waves and applying array signal processing techniques have been studied to overcome this limitation. Guided waves in a thin structure have the advantage of having a long wave propagation distance with small attenuation [13]. When a structural defect exists, it can be detected through the array signal processing technique by measuring the reflected wave from the ultrasonic transducer array. 

However, guided ultrasonic waves such as Lamb waves have multi-modes with difference phase speed at a same excitation frequency and this mixed response due to the multi-modes makes it difficult to apply the array signal processing techniques. Giurgiutiu [14] presented a mode-tuning technique by choosing an appropriate excitation frequency to excite a dominant single mode. Rose [15] suggested a single mode-excitation method by proposing comb transducers which could be locked to a specific wavelength. 

Yan et al [16]. applied a delay-and-sum beamforming algorithm to identify the defect location in plates. Han et al. found structural defects in an aluminum panel by constructing time-frequency MUSIC beamforming power maps and structural damping was considered to improve the spatial resolution [17]. These algorithms have a big advantage of excellent spatial resolution without the time-consuming scanning procedure, but they are difficult to apply to a real complex-shaped structure due to the numerous reflected waves and the complicated wave propagation characteristics. 

On the other hand, according to the demand for the effective fault diagnosis that can be applied to real complex-shaped structure, research on deep learning-based fault diagnosis has been actively conducted [18,19]. Among deep learning artificial neural networks, CNN (Convolutional Neural Network) is one of the effective algorithms in representing and extracting spatial patterns. Because of the efficiency and high accuracy in image classification, the CNN algorithm is widely used in plant disease diagnosis [20,21,22,23,24] and medical fields [25,26,27,28,29], as well as fault diagnosis of mechanical systems [30,31,32,33,34,35,36,37,38,39,40,41,42,43]. Typically, bearings are the biggest cause of motor failures, so plenty of CNN-based fault diagnosis studies have been conducted for monitoring the bearing condition [30,31,32,33,34,35,36,37,38]. Wang et al. conducted a study using three deep learning methods with data obtained by SCADA for fault diagnosis of power systems [39]. Zhong et al. applied SVM (Support Vector Machine) to identify small faults in the gas turbine by training changes in performance parameters such as exhaust gas temperature and fan speed when a fault occurred in a gas turbine [40]. In addition, researches have been conducted on detecting defects in composites using CNN training [41], determining defects in weld joints [42], and recognizing cracks in asphalt pavement [43].

As CNN is being actively used, studies have also been conducted to solve overfitting problems. Zheng et al. achieved a high level of classification performance by obtaining an initial distribution of samples through a pre-training process and detecting outliers through an implicit regularization training process to solve overfitting [44]. Ide et al. introduced sparseness to the input of rectified linear units to prevent unnecessary increase in the model parameters [45]. This can reduce overfitting and improve generalization by preventing unnecessary output of ReLU. EISayed proposed a regularization method called SD-Reg to improve network intrusion detection systems used to detect unseen intrusion events and solve overfitting [46]. This algorithm improved the performance over the existing L1 and L2 methods by using the standard deviation of the weight matrix. By solving overfitting of deep learning with these various methods, classification performance can be improved and deep learning can be comprehensively applied to various data.

In the case of the aforementioned beamforming algorithms, it is difficult to apply them when other sub-structures are attached to the specimen or when it has a complicated shape, since the reflected waves from the boundary can be mistaken for those from a defect. In order to solve this problem, in this study, a guided Lamb wave is excited on a plate with a defect and the measured wave reflected from the defect is 2D imaged. Then, CNN training is performed by labeling this image as the location of the defect. Even if an additional structure such as a stiffener is attached to the plate, as long as there is a difference in the measured data for each defect location, the defect location can be characterized by CNN feature extraction and it can be also applied to various mechanical systems.

As far as similar research, a study was conducted to apply the pattern of Lamb waves passing through defects on plates to the CNN algorithm [47]. However, this method has limitations on the number and location of defects due to the constrains that defects should exist between the actuator and the sensor. In this study, there is no such limitation, and the sensor array is arranged in the order of “actuator-sensor array-defect” to obtain the defect-induced reflected wave. In order to improve the defect-detection performance, the proper excitation position and sensor locations are investigated. For the efficiency of the experiment, the structural defect is simulated by attaching a coin [48], and difficulty in collecting a data set can be overcome since the simulated defect can be removed and attached easily.

In this study, an important factor that affects the accuracy of the results as much as the training data are the CNN architecture. Lecun et al. introduced the CNN in which the weights and biases of the convolution filter are automatically updated while reducing the error using the backpropagation method [49]. This network is called LeNet-1. As research based on it is actively conducted, LeNet-5 with an improved performance has been proposed [50]. In LeNet-5, the input data of size 32 × 32 passes through the convolution layer and the pooling layer twice, respectively, to create 16 feature maps of size 5 × 5. If this feature map is convolved with a 5 × 5 Kernel again, 120 feature maps of 1 × 1 size are created. All of them are connected to a fully-connected layer of size 84, and finally, when passing through an output layer of size 10, a training model with high performance can be obtained for the Modified National Institute of Standards and Technology database (MNIST) with class 10. Currently, numerous research on deeper and more effective neural networks such as AlexNet [51], VGG 16, VGG 19 [52], Goog-leNet [53], ResNet 18, ResNet 50, ResNet 101 [54], DenseNet 201 [55] are in progress. In this study, we tried to implement a CNN network for defect classification by referring to LeNet-5, which has the simplest structure.

## 2. Theory and Feasibility Study Using Simulation Model

### 2.1. Convolution Neural Network (CNN)

CNN is the most representative neural network used in deep learning that can be applied to image classification, object detection and tracking [56]. In this paper, CNN is used to characterize the reflected waves generated from defects in aluminum panels and classify related images. The architecture of the CNN algorithm is generally divided into two parts: (i) feature extraction and (ii) classification. 

#### 2.1.1. Feature Extraction

Feature extraction of the input image is performed by passing through layers composed of convolution, ReLU (Rectified Linear Units), and pooling. The convolution layer creates a feature map of the input image by performing the following operations [50]:

(1) $$Z_{i,j,k}^{l}={w_{k}^{l}}^{T}x_{i,j}^{l}+b_{k}^{l}.$$

where *l* and *k* are the index of the layer and filter, respectively, *i* and *j* are positions on the image, $w_{k}^{l}$ is the weight vector, $b_{k}^{l}$ is the bias term, $x_{i,j}^{l}$ is the input feature map, $Z_{i,j,k}^{l}$ is the output feature map. The following ReLU operation is performed to set all values smaller than zero to zero in the activation function to consider nonlinear features of the CNN [57].

(2) $$ReLU\left(Z_{i,j,k}^{l}\right)=max\left(0,Z_{i,j,k}^{l}\right).$$

Then, a pooling layer is applied to prevent overfitting and reduce the dimension of original image [51]. After downsampling to a rectangular pooling area, the maximum or average value for output is calculated.

#### 2.1.2. Classification 

After the feature map has been extracted, the output is flattened to a 1D vector and passed through a fully connected layer. The layer is fully connected to the *l*th layer as shown in the following Equation (3) multiplying the vectorized input value by the weight vector and adding the bias term. Then, the fully connected output is converted into a form of probability belonging to a specific class through softmax activation function [58].

(3) $$z_{i}^{l}=w_{ij}z_{j}^{l-1}+b_{i},$$

(4) $$Q\left(z\right)=\frac{exp\left(z_{j}\right)}{\sum_{j=1}^{K}exp\left(z_{j}\right)}.$$

Next, the cross-entropy loss function is computed as [30] follows:

(5) $$Loss=-\frac{1}{N}\sum_{i=1}^{N}\sum_{j=1}^{K}w_{j}t_{ij}lnlnq_{ij}.$$

where *N* and *K* are the number of samples and target classes, respectively, $w_{j}$ is the weigh vector for the *j*th target class, and $t_{ij}$ represents 1 if the *i*th sample belongs to the *j*th class, and 0 otherwise. $q_{ij}$ means the probability of connecting the *i*th sample to *j*th as a result of softmax function calculation. 

**Figure 1:**
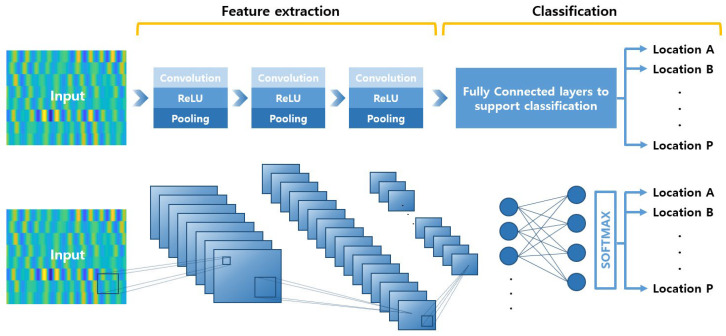
Convolutional Neural Network architecture for image classification.

When these series of layers are stacked, a network for image classification is prepared. The overall process of the CNN algorithm is schematized in Figure 1. A large amount of training data is essential for high classification performance, but there is a limit to obtaining sufficient data through experiments. Therefore, a deep learning network with a simple structure is used for the purpose of constructing an optimal transducer array and applying it to confirm the possibility of classifying defects [49,50]. 

### 2.2. Feasibility Study Using Simulation 2D Model

**Figure 2:**
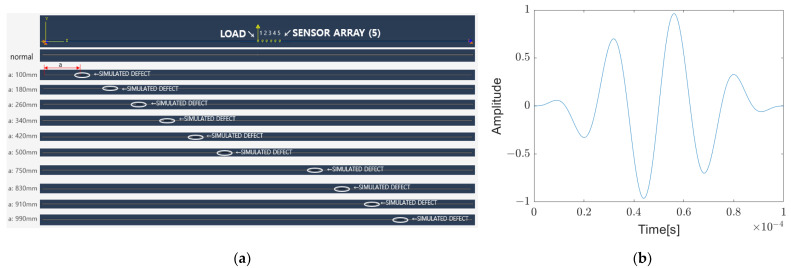
(**a**) Simulated 2D aluminum panels with defect; (**b**) Excitation signal with center frequency of 40 kHz.

**Figure 3:**
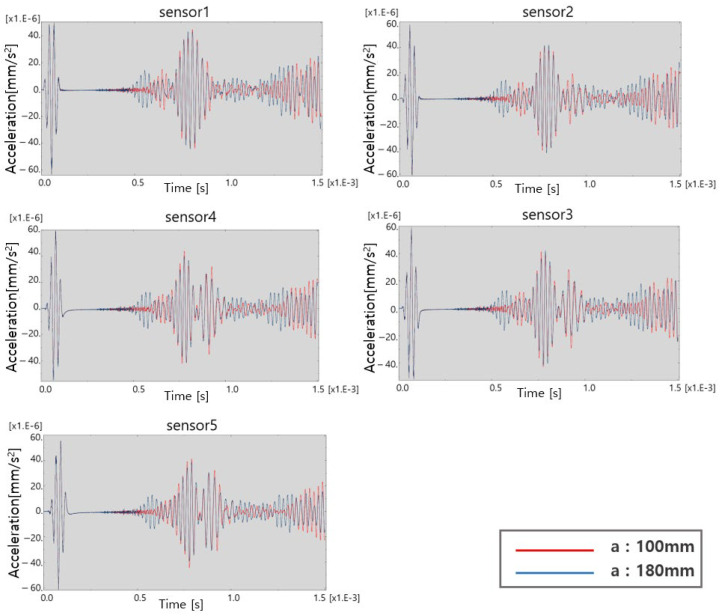
Measured results at each sensor location.

ABAQUS, a commercial finite element analysis software, is used for the feasibility study. As shown in Figure 2a, two-dimensional aluminum panels with defects equipped with five sensors and one actuator is modeled. The size of the aluminum panel is 1200 mm ⋅ 2 mm and the defect size is 20 mm × 1 mm. The center of the panel is excited with the burst sinusoidal signal shown in Figure 2b with the center frequency of 40 kHz. Excited elastic waves travel to both ends of the panel and are measured at the sensor array location. As shown in Figure 3, when comparing the two results obtained when a is 100 mm and 180 mm, respectively, where “a” is defined as the distance from the left end to the center of the defect, there is a significant difference in the measured signal depending on the location of the defect. It is confirmed that there is a possibility to identify the location of the defect. 

**Figure 4:**
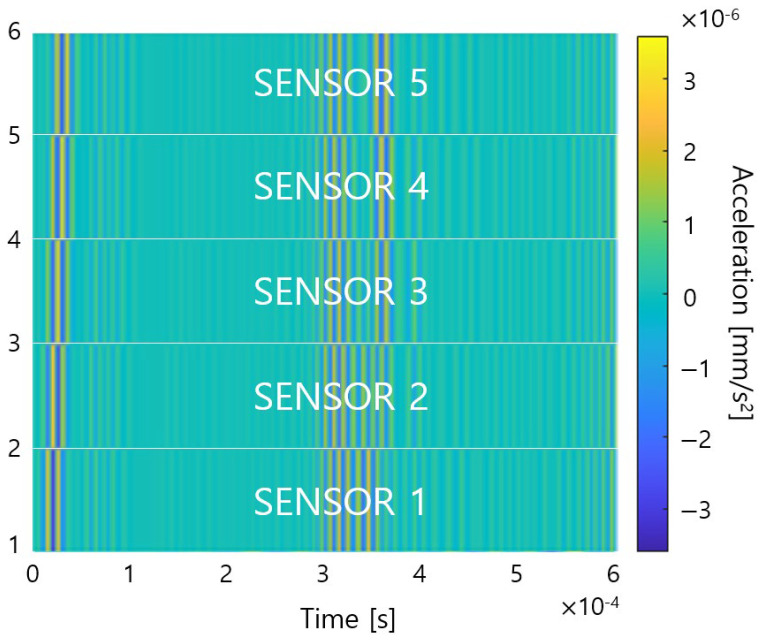
Example of converted image for training.

As mentioned in the previous chapter, CNN is a deep learning artificial neural network specialized in finding image patterns, so the measured data should be converted into a 2D image to identify the defect location. Therefore, different colors are applied according to the amplitude of the measured data, and the converted color bands are created as many as the number of sensors. Then, the color bands are layered and converted into one image as shown in Figure 4. The y-axis in Figure 4 means the number of stacked data, and the reason why the last value is 6 is because we added a zero vector in the matlab code to show the last color band.

**Figure 5:**
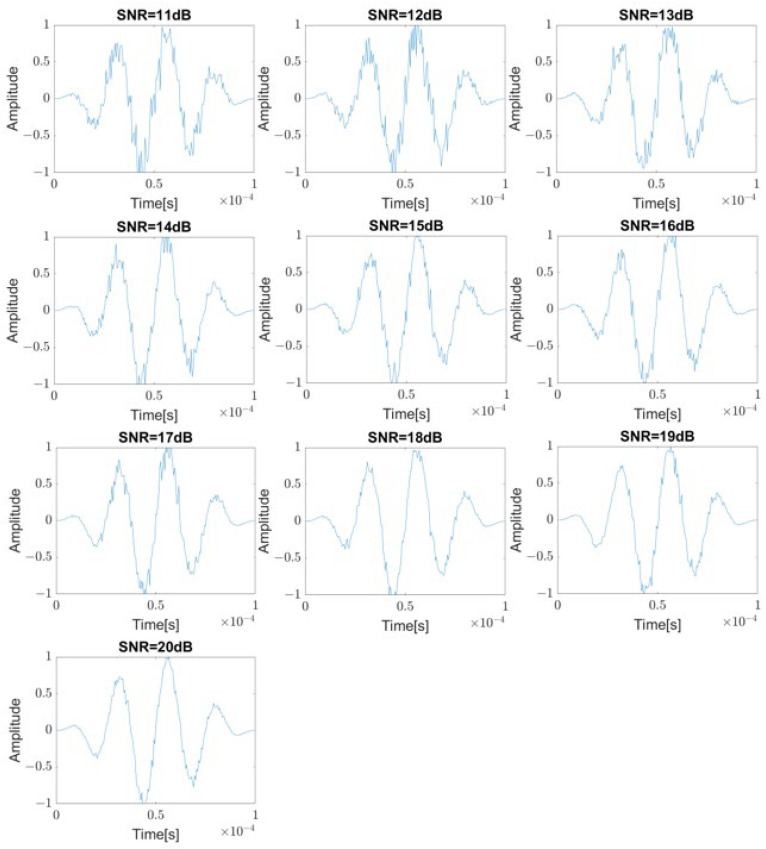
Excitation signal with center frequency of 40 kHz with SNR ranging from 11 dB to 20 dB.

In order to obtain an additional data set for training, 10 different images are acquired for each model by changing the SNR (Signal to Noise Ratio) of the excitation signal by 1 dB from 11 dB to 20 dB as shown in Figure 5. Each of these images is labeled with the location of the corresponding defect, and training is performed. The trained model shows 100% classification accuracy. For the purpose of checking the classification robustness, verification data are obtained using a model with a 5 mm error from the standard position. Contrary to expectations, the trained model determines the defect location to be totally incorrect instead of judging with the nearest position. It can be expected that the corresponding training model is overfitted and the error will be very large when applied to real cases. In order to solve this problem, from defects with ±2 mm and ±4 mm away from the standard positions, additional training data are obtained while changing the SNR of the excitation signal in the same way. After conversion to images, they are labeled as a defect from the standard position, and as shown in Table 1, 50 images for each label can be obtained. After training, the trained model successfully classifies the images obtained from defects with 3 mm and 5 mm away from the standard positions as the corresponding standard position, and the classification robustness can be achieved. 

## 3. Experimental Parameter Design Based on Finite Element Analysis

### 3.1. Excitation Frequency

The proposed algorithm can be applied without significant restrictions not only to the shape of the test object, but also to the excitation frequency or ultrasonic mode if there is a difference in the reflected wave by a defect depending on the defect location. In Section 2.2, the trained image obtained from the defect-reflected wave can determine the defect location; therefore, ensuring that the reflected signal does not overlap with other signals will make it easy to characterize the defect location in an experimental way. 

**Figure 6:**
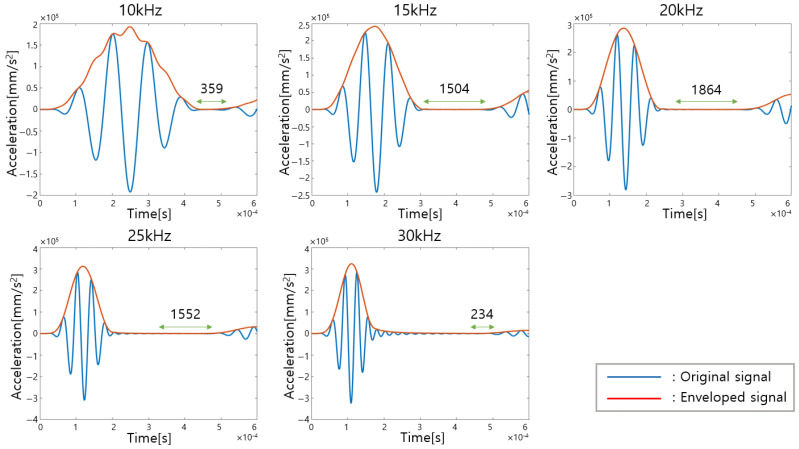
Time interval between direct incident wave and boundary-reflected wave according to excitation frequencies.

The center of a defect-free aluminum panel with a 2 mm thickness is excited from 10 kHz to 30 kHz, and the response at a distance of 30 mm is calculated through a finite element simulation. The time interval from the arrival of all direct-excitation waves to the point at which the reflected waves from the boundaries just begin to arrive is considered as the interval at which the reflected wave from a defect can be measured. In order to compare the time interval length according to the excitation frequency, the corresponding measured data are enveloped as shown in Figure 6. When the excitation frequency is 20 kHz, it is confirmed that the time interval estimated to measure the defect-reflected wave is longest. 

### 3.2. Excitation Location

**Figure 7:**
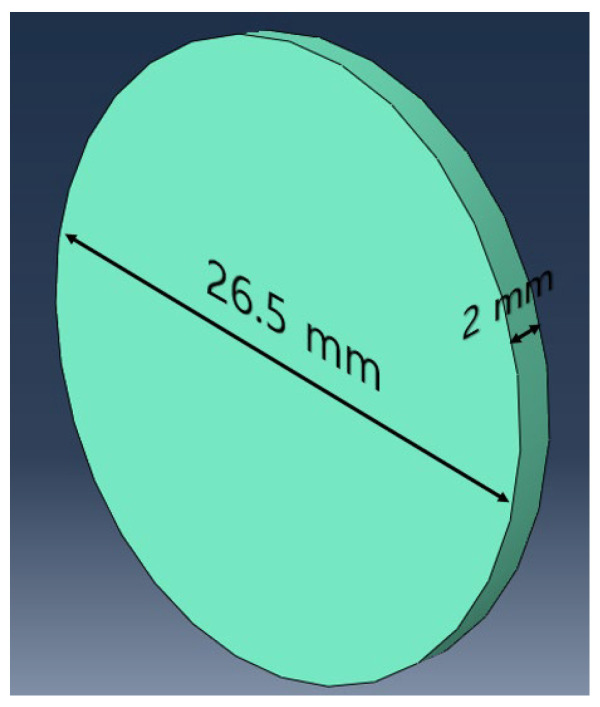
Dimension of defect in FE simulation.

**Figure 8:**
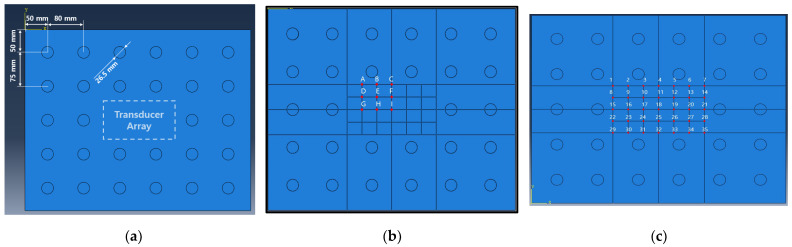
(**a**) Simulated circular defects on aluminum panel; (**b**) candidate excitation locations; (**c**) sensor locations.

**Figure 9:**
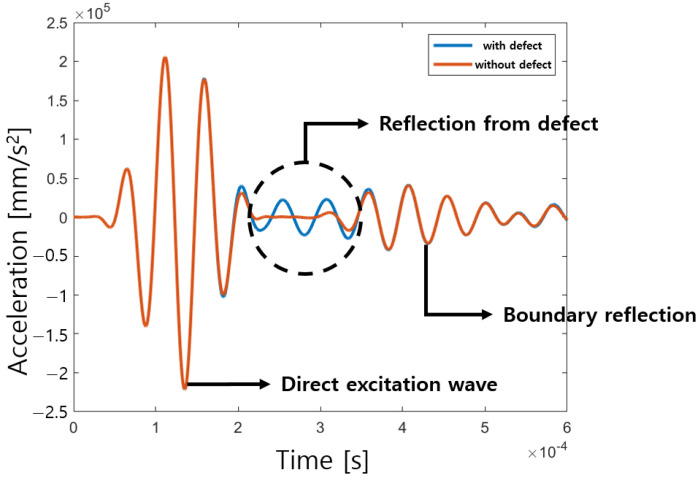
Time responses of panel with and without defect at 20 kHz excitation.

In this chapter, optimal excitation location is determined. The defect is modeled as shown in Figure 7 and Table 2. It is classified into 28 positions as shown in Figure 8a. When the panel is excited at each candidate position shown in Figure 8b, the excitation position where the amplitude summation of the reflected wave from each defect location becomes maximized is selected. The detailed procedure is as follows. In one of the excitation candidates, excite the panel with the excitation frequency of 20 kHz and calculate the response measured at the 35 sensors in Figure 8c. This process is repeated as many as 28 defect positions and nine potential excitation locations (i.e., 28 × 9 = 252 times). For the efficient CNN training, as shown in Figure 9, only the reflected signal caused by the defect is extracted by calculating the difference between the result obtained from the defect-free model and from the model with a defect. Then, by doing the sum of all magnitudes of the reflected waves obtained from the 28 positions, the position B is determined as the optimal excitation position that can generate the largest defect-reflected wave as shown in Table 3. 

**Figure 10:**
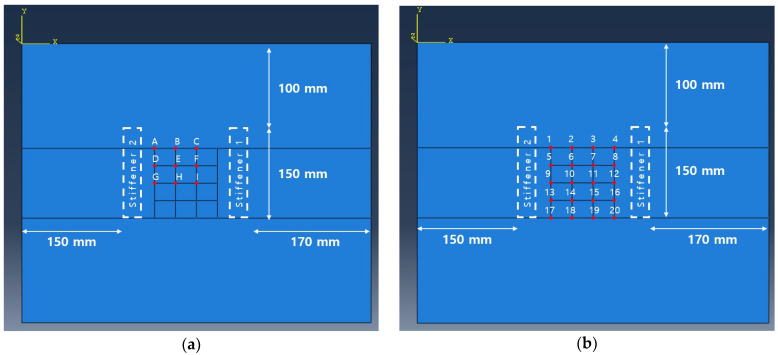
(**a**) Candidate excitation locations on aluminum panel with stiffener; (**b**) sensor locations on aluminum panel with stiffener.

For the cases where a stiffener is attached to each left and right side of the transducer array respectively, it is modeled as shown in Figure 10 to determine the optimal excitation location. In order not to overlap the position of the attached stiffener and the sensor position, the existing 35 sensors are changed to 20 sensors and it is simulated in the same way as above. As shown in Table 4, as a result of summing up the magnitudes of reflected waves from 28 defect positions, it is found that it is most efficient to place the actuator at position B when the stiffener is on the right side of the sensor array. Plus, position A is determined as the optimal excitation position when the stiffener is on the left side of the sensor array as shown in Table 5.

### 3.3. Sensor Location

**Figure 11:**
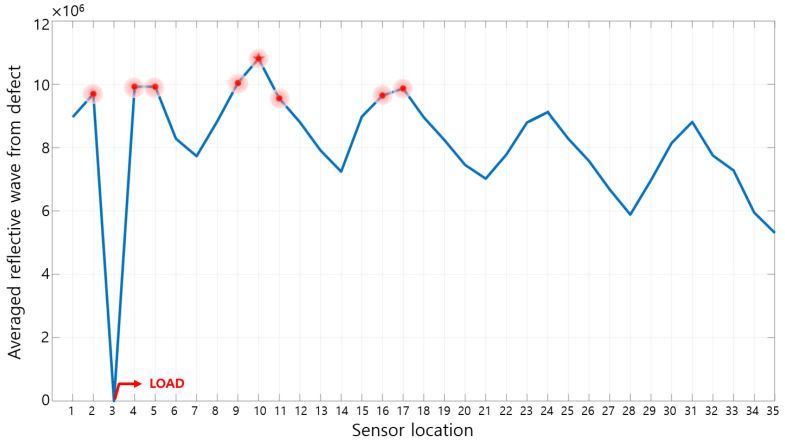
Averaged magnitude of the reflective waves from defects measured by each sensor.

**Figure 12:**
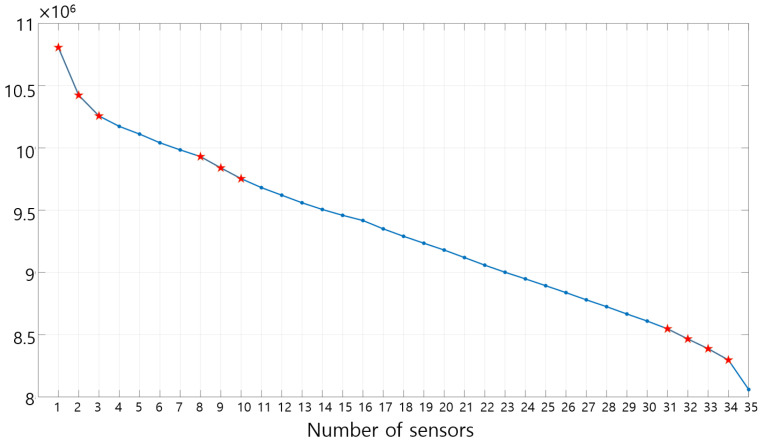
Averaged magnitude of the reflected wave from ‘one’ sensor (rearranged in order from the largest value of the averaged amplitude).

The sensor position where the reflected wave from the defect can be observed most effectively is investigated. In order to quantitatively evaluate the averaged amplitude of the reflected wave measured by the sensors, the calculation process of Equation (6) is performed. Using Equation (6), the averaged amplitude of the reflected wave according to each sensor location is shown in Figure 11, and the data with relatively large values are marked with red dots to select the sensor position to be used in the experiment. As a criterion for determining the number of sensors, the averaged amplitude of the reflected wave from the defect is rearranged in order of the averaged amplitude, and then cumulatively summed and added in order from the largest value and divided by the number of added values as shown in Figure 12. In Equation (7), *a_n_* means the *n*-th largest averaged amplitude of the reflected waves measured for each sensor, and *x_k_* is the expected, averaged magnitude of the reflected wave from ‘one’ sensor when the number of sensors is *k*. Figure 12 shows *x_k_* and the 10 positions where the slope of the graph changes the most are marked with red asterisks. Excluding the extreme number of sensors in Figure 12, the eight to 11 sensors are considered effective. The purpose of this study is to characterize the defect location with a small number of sensors, so the number of sensors is set to eight.

(6) $$Averaged\;reflective\;wave\;from\;defect=\frac{\sum abs\left(Reflective\;wave\;from\;defect\right)}{Number\;of\;defect\;locations}.$$

**Figure 13:**
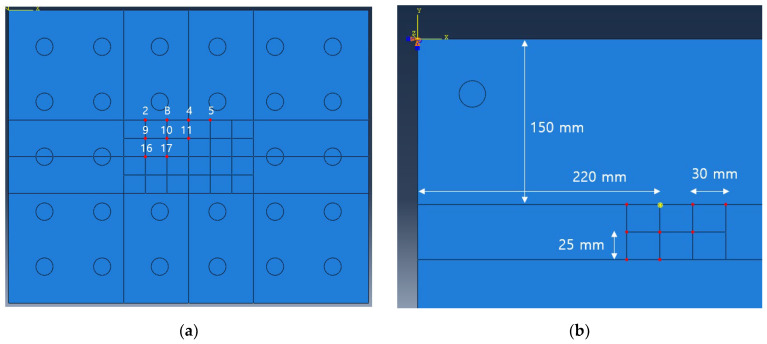
(**a**) Optimal transducer array configuration; (**b**) detailed dimensions of transducer array on aluminum panel.

**Figure 14:**
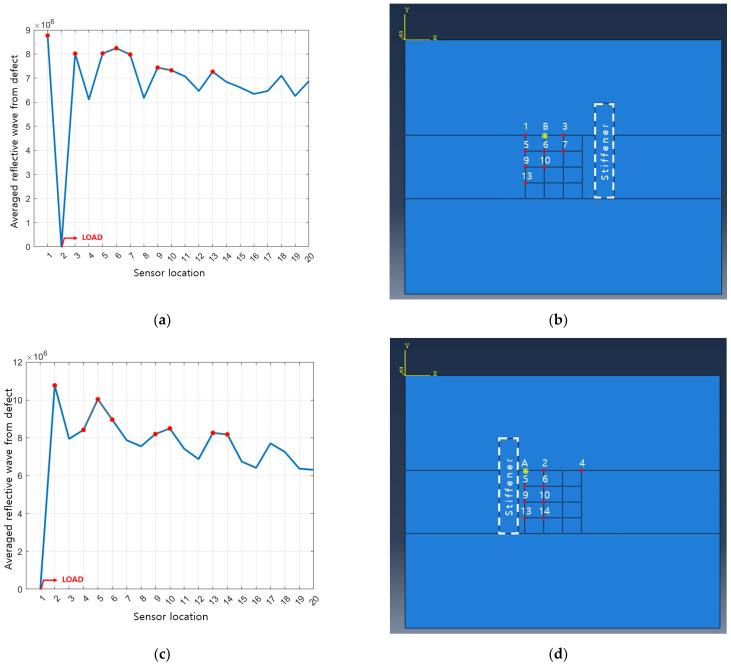
(**a**) Averaged magnitude of the reflective waves from defects measured by each sensor where stiffener is attached on the right side of panel; (**b**) optimal transducer array configuration where stiffener is attached on the right side of panel; (**c**) averaged magnitude of the reflective waves from defects measured by each sensor where stiffener is attached on the left side of panel; (**d**). optimal transducer array configuration where stiffener is attached on the left side of panel.

Figure 13 shows the selected excitation position and sensor position. In the same way, when the stiffener is attached to the aluminum panel, the excitation location and the sensor locations are determined as shown in Figure 14. As a result, it is possible to efficiently implement a transducer array capable of observing the reflected waves from defects with a relatively small number of transducers.

(7) $$x_{k}=\frac{\sum_{n=1}^{k}a_{n}}{k}.$$

where *a_n_* is the *n*-th largest averaged amplitude of the reflected waves measured for each sensor.

## 4. Experimental Setup and CNN Training Procedure

### 4.1. Experimental Setup

**Figure 15:**
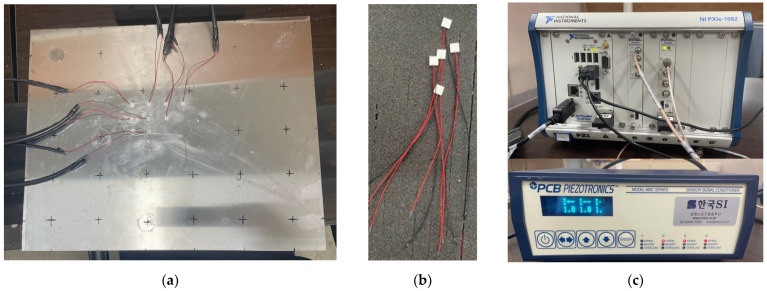
Photos of experimental setup: (**a**) transducer array and defect attached on aluminum panel; (**b**) piezoelectric transducer (7 mm × 7 mm, APC International, Ltd., Mackeyville, PA, USA); (**c**) NI DAQ system equipped with waveform generator (NI-PXIe-5423) and oscilloscope module (NI-PXIe-5172) and signal conditioner (482C24, PCB PIEZOTRONICS Inc., Depew, NY, USA).

**Figure 16:**
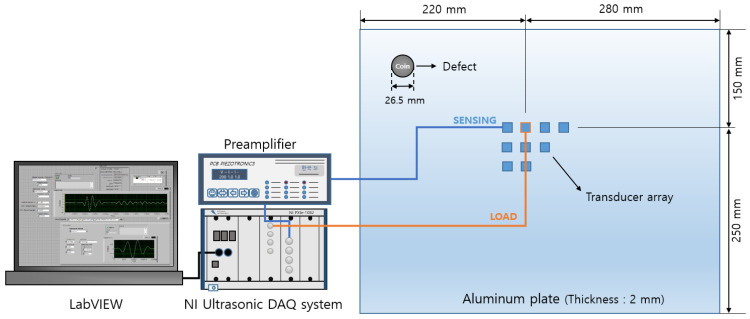
Schematic sketch of experimental setup.

The extracted information about the excitation frequency and optimal transducer locations are applied to an experiment setup to obtain a data set for CNN training. In order to efficiently collect data for each defect at various locations on the panel, the structural defect is simulated by attaching a coin with a diameter of 26.5 mm, a thickness of 2 mm, and a weight of 7.7 g to the aluminum panel. The method of attaching coins or mass blocks to simulate structural defects has also been used in previous studies [17,45], and this method allows the defect to be easily removed or attached. Therefore, it can save time and costs rather than making permanent notches or cracks in the panel surfaces. After attaching a coin, at the sensor locations, eight piezoelectric transducers are attached on the aluminum panel using a superglue and a transducer is also attached at the excitation location as shown in Figure 15a,b. A National Instruments (NI) system equipped with a signal generator module and an ultrasonic data acquisition module is used to generate a 20 kHz Lamb wave and measure the waves amplified by a signal conditioner as shown in Figure 15c. Figure 16 shows a schematic diagram of experimental setup. 

### 4.2. Data Acquisition

**Figure 17:**
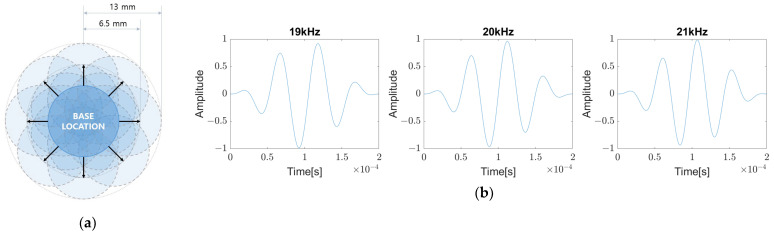
(**a**) Multiple defect locations to train for one standard position; (**b**) excitation signal for each frequency.

To increase the training data set, similarly to the method mentioned in Section 2.2, data are obtained while moving the coin twice by 6.5 mm and 13 mm in eight directions from the standard position as shown in Figure 17a. At the same time, by changing the excitation frequency to 19 kHz, 20 kHz, and 21 kHz, respectively, as shown in Figure 17b, 51 (=17 × 3) data from a sensor for each defect are collected and a total of 408 (=51 data/sensor × 8 sensors) data are gathered. 

**Figure 18:**
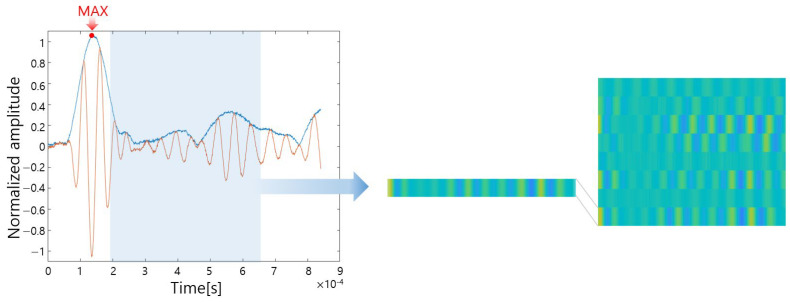
Roughly measured time of the reflective waves from defects and converted training image based on the time interval.

On the other hand, it is analyzed that the reflected wave from a defect is measured approximately from 0.06 ms to 0.52 ms after reaching the maximum value of the direct wave. In order to extract the features, the corresponding time data of the defect-induced reflected wave at each sensor are converted into a color band. Then, a 2D image is created by stacking the color bands upward as shown in Figure 18. In this way, 51 data sets are obtained for each defect, and all of them are labeled as the reference defect located at the corresponding standard position. Among them, the image obtained from the defect in the middle position, excited at 20 kHz, is used as a verification image, and CNN training is conducted with the remaining 50 images. MATALB is used for the image creation and CNN training and validation.

### 4.3. CNN Training

#### 4.3.1. Simply Designed Network

The size of the input image is 539 × 682 × 3, and the initially designed convolution kernel is composed of three two-dimensional layers. The size of all filters is the same as 5 × 5, and the number of the filter is 8, 16, and 32, respectively. A 5 × 5 filter scans the input with a stride of 1. After the first convolution, the size is reduced to 535 × 678 × 3, but the zero padding is applied to match the input size. The batch normalization layer, located after the convolutional layer, speeds up neural network training and stabilizes learning. As a nonlinear activation function, the commonly used ReLU function is used for fast computation and high accuracy, and a 2 × 2 rectangular filter scans the input value with a stride of 2 and it goes through a maxpooling process that returns the maximum value. After being downsampled in this way, it is input into the next convolution layer and the above process is repeated. After feature extraction, it is input into the last fully connected layer, and the loss is calculated through the SoftMax layer and the input value is predicted for each class. These processes are shown in Figure 1. 

**Figure 19:**
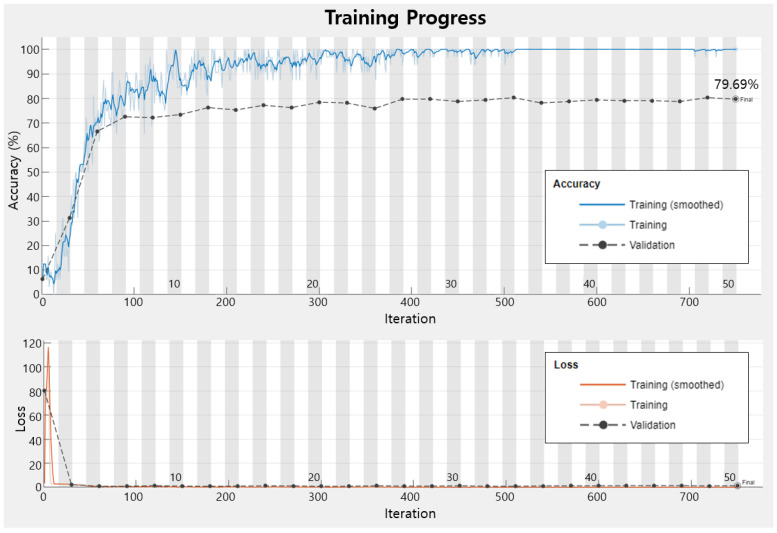
Convergence curve of CNN model with 50 epochs.

However, if the experiment is conducted with only one network, the performance of the training model cannot be accurately evaluated. On the other hand, variables that can be modified in the convolution layer can be the size of the kernel, the number of kernels, and the number of layers. Therefore, the rationale for the size and number of convolution kernels and the number of layers should be explained and determined. Among them, considering that there are less than 100 training data, the number of layers is fixed to a relatively small value of 3 and the optimal kernel size and number are selected under the condition that the number of kernels increases by a factor of two. Hence, the number of possible cases is 16 (= 4 × 4) shown in Table 6. The size of the kernel is set to an odd number to maintain the symmetry of the image in the zero-padding layer following the convolution layer, and the number of kernels is set to 2n for efficient computation on the GPU. The training data set is obtained by setting the defect candidate positions on the bare panel to be 16 classes and the training conditions are all the same as Table 7. Max epoch is trained 50 times, which is considered that the accuracy and loss converged in all cases as shown in Figure 19. If the number of kernels is large, many features can be extracted, but the learning speed becomes slow and overfitting can occur. Therefore, it should be determined by considering the learning time, testing accuracy, and validation accuracy [59]. 

According to the training results summarized in Table 6, as the number of kernels increases, the classification accuracy increases and the training speed decreases. When the number of kernels is 32/64/128, it is considered to be inefficient because the training time is more than doubled compared to the increase in accuracy. In addition, the training speed becomes slow and the classification accuracy is low when the filter size is 7 × 7 and 9 × 9 compared to 3 × 3 and 5 × 5, so it is considered to be unsuitable. Excluding the inappropriate cases, the remained six cases are verified with data that do not participate in the training to determine the accuracy in Table 8. As a result, the parameters with the highest accuracy (i.e., size: 3 × 3, number: 16/32/64) are selected, and a modified convolution layer is developed according to the conditions.

**Figure 20:**
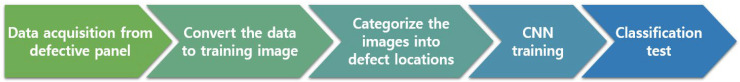
CNN training process for classification.

The CNN training options are set as shown in Table 7 through the trial-and-error method. As a solver in this study, stochastic gradient descent with momentum (SGDm) which is an improved version of SGD is used. By adding the momentum term to the SGD solver, the calculation speed is faster than SGD and it allows better convergence to the global minimum point by preventing the trapping of local minima. The inertia coefficient of SGDM is set to 0.9, which is generally used. Figure 19 shows the convergence curve of the designed CNN model and the entire training process is shown in Figure 20.

#### 4.3.2. Pre-Trained Network

The simple convolution network in the previous sub-section has the advantage that the layer parameters can be easily modified and supplemented to achieve the classification performance, but the number of possible combinations is too big, making it difficult to design a network architecture with the best classification performance. In addition, overfitting easily occurs when the convolutional layers are stacked too much in order to improve classification performance, and because of this risk, overfitting must be checked with verification data. A pre-trained network can be useful when data acquisition is limited or when a high-performance training model is required in a short time since it can apply the weights obtained by already training more than one million images to new training. Therefore, it can be a suitable training model that can reduce the risk of overfitting under this condition where training data is not sufficient. For this reason, nine commonly used pre-trained networks are used to apply the experimental data to transfer learning and to check their performance as shown in Table 9. The input image is resized to the size required by each network, and the fully connected layer and classification layer are modified according to the number of output classes, and then training is performed. The training conditions are shown in Table 8, and the accuracy of the training results is shown in Table 9. Two architectures with the highest accuracy are identified as ResNet50 and DenseNet201.

#### 4.3.3. Comparison of Networks

**Figure 21:**
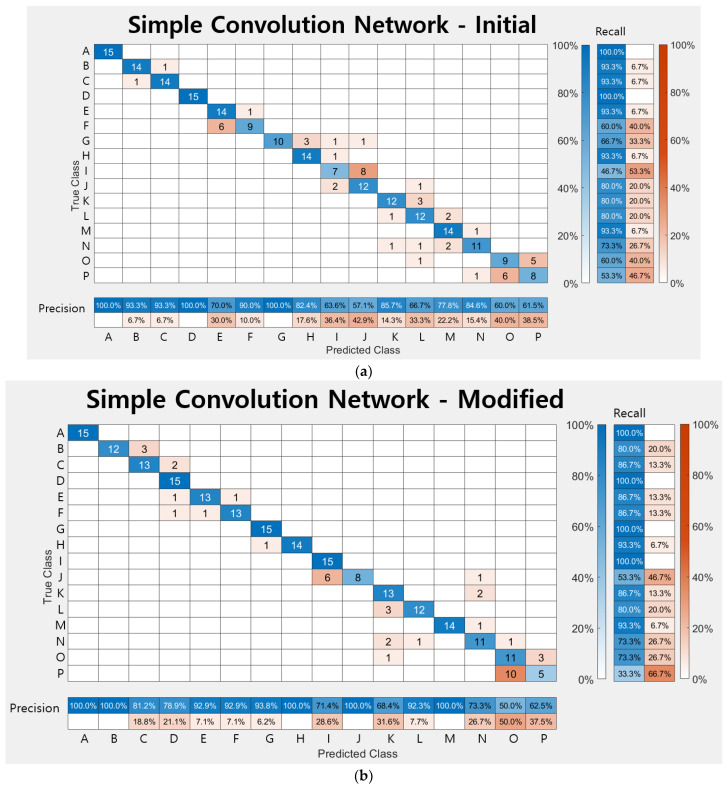

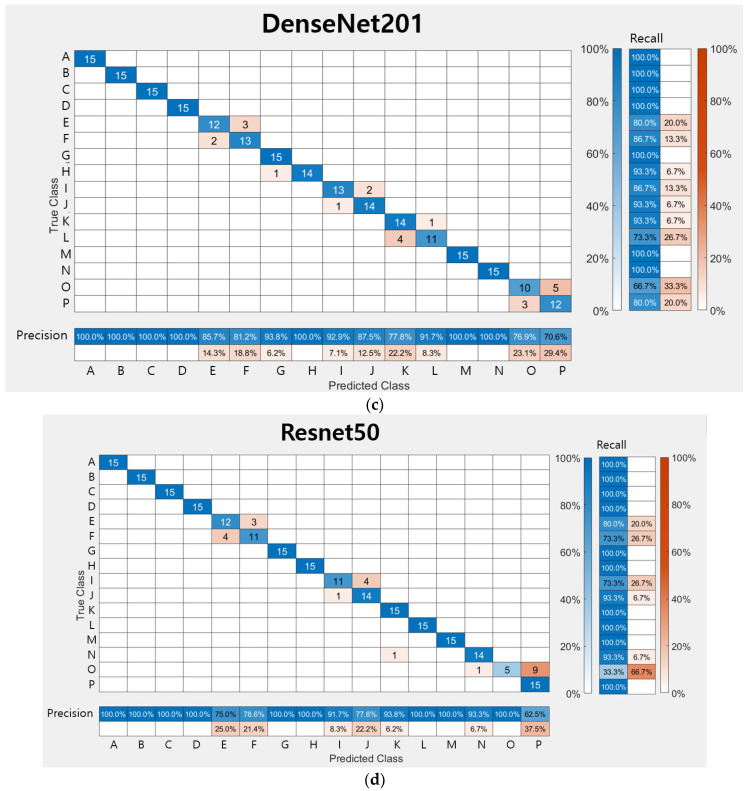
Confusion charts: (**a**) Initially designed simple network; (**b**) Modified simple network; (**c**) DensNet 201 and (**d**) ResNet50.

**Figure 22:**
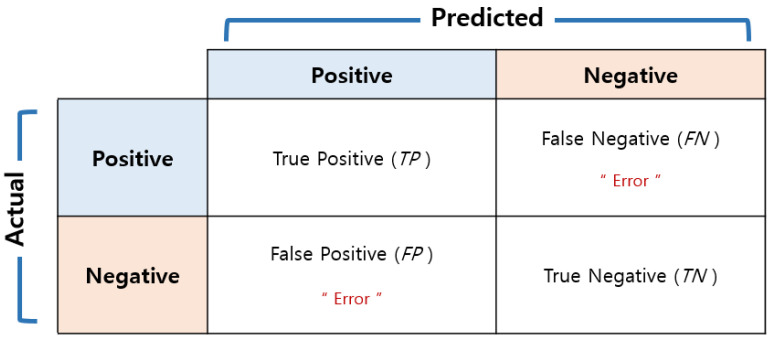
Performance metrics for classification.

Through the Section 4.3.1 and Section 4.3.2, the four networks for this experiment are compared with each other. In order to check the accuracy of each class, a confusion chart with a size of 16 × 16 is created based on the class prediction result and the actual label value as shown in Figure 21. Since the training in this study is multi-class rather than binary class, the output is not divided into 0 and 1, the case where the verification data are classified into the corresponding class for each class is set as 1, and the other case where it is not is set as 0. The ‘precision’ displayed on the horizontal axis in Figure 21 means the ratio of data corresponding to actual positive among the predicted positive data as shown in Equation (8) and Figure 22, and the ‘recall’ on the vertical axis means the ratio of actual positive predicted as positive as described in Equation (9). The accuracy is calculated using Equation (10) and the F1 score is the harmonic average of precision and recall, as expressed in Equation (11), and is used when considering the two conflicting metrics. As shown in Table 10, when comparing the four networks, the F1 scores of transfer learnings are about 10% higher than that of the simply designed network, and DenseNet201 has the highest F1 score.

(8) $$Precision=\frac{TP}{\left(TP+FP\right)},$$

(9) $$Recall=\frac{TP}{\left(TP+FN\right)},$$

(10) $$Accuracy=\frac{TP+TN}{\left(TP+TN+FP+FN\right)},$$

(11) $$F1\;score=2*\frac{Precision*Recall}{Precision+Recall}$$

## 5. Result and Discussion

In order to evaluate the influence of elements in the designed network, the ablation test and comparison test are performed as shown in Table 11 and Table 12. For the purpose of investigation on the effect of the changes in the defect location and the excitation frequency, the classification accuracy for each location is calculated by verifying with data that do not participate in the training after 10 training sessions. To simplify the comparison, only six out of 16 defect cases are trained.

**Figure 23:**
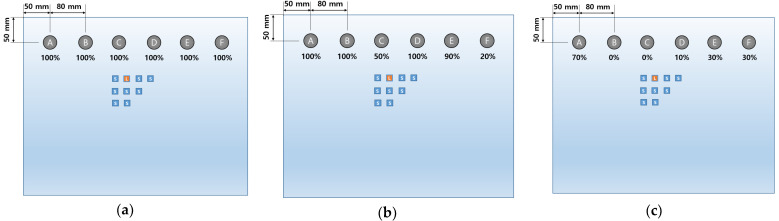
(**a**) Classification accuracy for six defect locations which is trained by whole data set; (**b**) classification accuracy for six defect locations. The training data set is collected by shifting location of defect data sets; (**c**) classification accuracy for six defect locations. The training data set is collected by shifting location of defect data sets.

First, when training is conducted 10 times with 50 full data sets and verified with one datum, 100% accuracy is observed in all locations as shown in Figure 23a. Next, in order to investigate the effect of the excitation frequency change on the training data set, training and verification are performed with only 17 data excited at 20 kHz, excluding data with the excitation frequencies of 19 kHz and 21 kHz. At this time, it is verified with data obtained from the defect existing at the standard position and trained with the remaining 16 images. As a result, as shown in Figure 23b, a little lowered accuracy is observed at the positions C, E, and F. In addition, in order to look into the effect of changing the defect location on the training data set, training is conducted with only three data acquired from the standard location. Due to lack of training data, it is trained using 15 overlapped data and verified with data located 6.5 mm (i.e., about 1/2 of the defect radius) away from the standard position. The corresponding result is shown in Figure 23c. Although the verification accuracy during training is 100%, when it is verified with new data, the accuracy becomes very low at most positions, indicating that the training model is overfitting.

Through these tests, it can be observed that the change in defect location has a greater effect on the training result than the change in the excitation frequency. Therefore, acquisition of the additional training data set while slightly moving the defect position is essential to obtain a robust training model, and it can be concluded that changing the location of the defect is more effective than changing the excitation frequency as a way to increase the data set.

**Figure 24:**
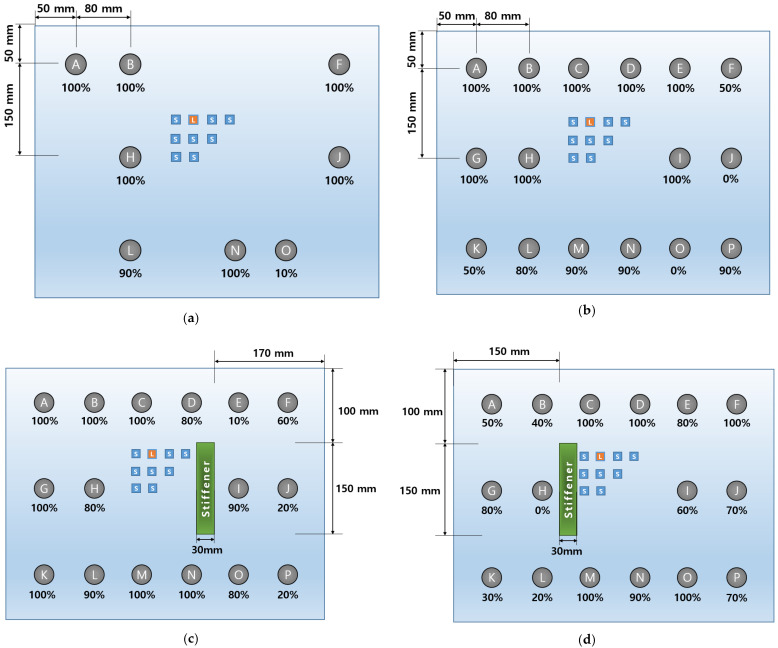
(**a**) Classification accuracy for randomly selected eight defect locations; (**b**) classification accuracy for 16 defect locations; (**c**) classification accuracy for attaching steel bar to right side of panel; (**d**) classification accuracy for attaching steel bar to left side of panel.

As shown in Figure 24a, the classification possibility is investigated for eight randomly selected positions on the aluminum panel. A total of 400 images ( = 50 × 8) obtained from experiments are labeled with eight classes, respectively, and 10 training models are derived by training 10 times. As a result of classifying the verification image which is excluded from training, the classification accuracy for each location is calculated as shown in Figure 24a. The average classification accuracy for the eight locations is 87.5%. 

The classification performance was successfully verified in the eight-defects case. As shown in Figure 24b, the number of defect locations is expanded to 16. A total of 800 images are used as a data set by conducting the experiment under the same conditions. It is labeled as 16 classes, respectively, and 10 training models are obtained. Even though the number of defect candidates doubles, 12 out of 16 locations shows classification accuracy of 80% or more. The average classification accuracy for 16 locations is 78.1%, showing a decrease in accuracy of 9.4% compared to the previous case. It is observed that the classification accuracy of some positions decreases as the number of defect candidates increases. When DenseNet201 is applied through the F1 score summarized in Table 10, it is expected that classification will be possible at the ‘J’ or ‘O’ position, which is difficult to classify in Figure 24b.

Even if a stiffener is additionally attached to the test object, the presented algorithm in this paper is expected to be able to extract features with a small difference in the measured signals on the sensor array. In this case, non-destructive testing using the beamforming methods [16,17] becomes very difficult to apply since the steering vector, that is a kind of spatial transfer function, is disturbed by the additional structure. To experimentally verify this case, a 5 mm thick steel bar is attached to the panel while maintaining all conditions, and the experiment is conducted. For this case, in order to investigate the classification possibility of defects and the change in classification accuracy according to the attachment of stiffeners, as shown in Figure 24a and Figure 24b, experiments are conducted under the same transducer-placement conditions as without stiffeners. The steel bar is attached to the right side of the transducer array as shown in Figure 24c. As a result of verification, the classification accuracy of over 80% is shown for 12 out of 16 locations. Table 13 summarizes the average classification accuracies. The classification accuracy of the left side of the panel increases by 6.6% above the average compared to when there is no bar while the classification accuracy of the right side decreases by 8.8%.

Next, as shown in Figure 24d, the steel bar is attached to the left side of the panel. When the bar is attached to the left side of the panel, the classification accuracy on the left side is significantly lowered to 52.5% while the classification accuracy on the right side increases by 17.5% compared to when there is no bar. This can be inferred from the fact that the location of the defect may not be accurately classified if there is an interfering structure such as a stiffener between the reflected wave and the sensor array. 

In order to solve this problem, if there is an arbitrary structure in the test structure, a method of additionally placing sensors on both sides of the structure can be considered. In this study, we mainly dealt with the optimization of the sensor locations for a bare panel, and since there is a limitation to collect the data set through the experiment, only the classification possibility could be known. In the future, if a larger data set can be obtained using various methods, it is expected that the classifiable area can be improved with a high classification accuracy.

## 6. Conclusions

In this study, a method using a CNN algorithm was presented to classify the location of defects in a panel. The characteristics of reflected waves from a defect was extracted by applying the CNN algorithm. The excitation frequency of the experiment was set to 20 kHz in consideration of the time interval in which the reflected wave from the defect was converted into a 2D image. The optimal excitation position was selected as the position where the magnitude of the reflected wave measured at sensor candidate positions was the largest. In addition, the sensor locations were selected as the positions where the largest magnitude of the reflected wave could be measured. 

In order to create a training data set, the experiment was conducted by collecting 51 data for one standard position while slightly changing the location of the deflect and the excitation frequency. A training data set was obtained from eight randomly selected position and its average classification accuracy of 87.5% was achieved. Then, the number of defect candidates was increased to 16, resulting in the average classification accuracy of 78.1%. When the bar was attached to the right side of the panel, the average classification accuracy increased by 6.6% at the left side of the panel while it decreased by 8.8% at the right side of the panel. When the bar was attached to the left side of the panel, the average classification accuracy increased by 17.5% at the right side of the panel while it decreased by 37.5% at the right side of the panel. In the future, if a larger data set can be obtained using various methods, it is expected that the classifiable area can be improved with a high classification accuracy. 

## Figures and Tables

**Table 1:** Expanded data set labeled with 10 classes based on the defect locations.

Label	Size of Data Set
a_100 ^1^	50
a_180	50
a_260	50
a_340	50
a_420	50
a_500	50
a_750	50
a_830	50
a_910	50
a_990	50

^1^ ‘a:100’ in Figure 2a labeled as ‘a_100’, The same applies to the rest.

**Table 2:** Material properties of defect in FE simulation.

Density	Young’s Modulus	Poisson’s Ratio
7.00 × 10^−3^ g/mm^3^	150 GPa	0.32

**Table 3:** Summed magnitudes of defect-induced reflective waves for each excitation location.

Location of Excitation	Magnitude of Reflective Wave from Defect
B	1.35 × 10^8^
E	1.29 × 10^8^
I	1.28 × 10^8^
F	1.27 × 10^8^
H	1.27 × 10^8^
G	1.25 × 10^8^
C	1.24 × 10^8^
D	1.23 × 10^8^
A	1.22 × 10^8^

**Table 4:** Summed magnitudes of defect-induced reflective waves for each excitation location where stiffener is attached on the right side of panel.

Location of Excitation	Magnitude of Reflective Wave from Defect
B	2.95 × 10^8^
H	2.84 × 10^8^
E	2.83 × 10^8^
C	2.78 × 10^8^
I	2.77 × 10^8^
F	2.76 × 10^8^
G	2.76 × 10^8^
D	2.69 × 10^8^
A	2.66 × 10^8^

**Table 5:** Excitation locations as function of summed magnitudes of defect-induced reflective waves where stiffener is attached on the left side of panel.

Location of Excitation	Magnitude of Reflective Wave from Defect
A	1.50 × 10^8^
B	1.47 × 10^8^
D	1.42 × 10^8^
E	1.36 × 10^8^
G	1.36 × 10^8^
H	1.34 × 10^8^
C	1.27 × 10^8^
F	1.23 × 10^8^
I	1.23 × 10^8^

**Table 6:** Elapsed time and validation accuracy for CNN training by different size and number of kernels.

	Number	4, 8, 16	8, 16, 32	16, 32, 64	32, 64, 128
Size					
3×3	Time [s]	301	352	424	1580
	Accuracy [%]	78.33	78.75	82.92	83.33
5×5	Time [s]	246	348	510	1821
	Accuracy [%]	78.75	79.17	83.33	85.00
7×7	Time [s]	311	413	670	1558
	Accuracy [%]	75.42	72.08	81.25	81.67
9×9	Time [s]	322	428	696	1625
	Accuracy [%]	76.67	76.67	81.25	84.58

**Table 7:** CNN training options.

Total Number of Data	Training Data	Testing Data	Optimizer	Mini Batch Size	Initial Learn Rate	Shuffle
50	35	15	SGDM	32	0.01	Every epoch

**Table 8:** Testing accuracy.

	Number	4, 8, 16	8, 16, 32	16, 32, 64
Size				
3×3		70	70	80
5×5		50	60	70

**Table 9:** Transfer learning results.

Architectures	Depth	Image Input Size	Initial Learn Rate	Accuracy (%)
AlexNet [51]	8	227 × 227 × 3	3 × 10^−4^	82.08
VGG 16 [52]	16	224 × 224 × 3		80.83
VGG 19 [52]	19	224 × 224 × 3		81.25
GoogleNet [53]	22	224 × 224 × 3		79.58
ResNet 18 [54]	18	224 × 224 × 3		84.58
ResNet 50 [54]	50	224 × 224 × 3		90.42
ResNet 101 [54]	101	224 × 224 × 3		86.25
DenseNet 201 [55]	201	224 × 224 × 3		90.83

**Table 10:** Calculated F1 Score (%).

	Architecture	Initial Simple Network	Modified Simple Network	DenseNet201	ResNet50
Output Classes					
A		100	100	100	100
B		93.33	88.89	100	100
C		93.33	83.87	100	100
D		100	88.24	100	100
E		80.00	89.66	82.76	77.42
F		72.00	89.66	83.87	75.86
G		80.00	96.77	96.77	100
H		87.50	96.55	96.55	100
I		53.85	83.33	89.66	81.48
J		66.67	69.57	90.32	84.85
K		82.76	76.47	84.85	96.77
L		72.73	85.71	81.48	100
M		84.85	96.55	100	100
N		78.57	73.33	100	93.33
O		60.00	59.46	71.43	50.00
P		57.14	43.48	75.00	76.92
Average		78.92	82.60	90.73	89.79

**Table 11:** Ablation test result.

Model	Full Model (No Stiffener)	Shifting Location of Defect	Changing Excitation Frequency
**Accuracy**	0.7917	1.000	0.7396

**Table 12:** Comparison test results.

Model	Full Model (No Stiffener)	Stiffener Attached	
		Steel	PLA
**Accuracy**	0.8625	0.4625	0.5333

**Table 13:** Averaged classification accuracy.

Figure Number	State of Panel	Classification Accuracy (%)		Total Averaged Accuracy (%)
24a	Without bar	Left	97.5	87.5
		Right	77.5	
24b	Without bar	Left	90.0	78.1
		Right	66.3	
24c	With bar on right	Left	96.6	76.9
		Right	57.5	
24d	With bar on left	Left	52.5	68.1
		Right	83.8	

## Data Availability

Not applicable.
